# FBXL19-AS1 exerts oncogenic function by sponging miR-431-5p to regulate RAF1 expression in lung cancer

**DOI:** 10.1042/BSR20181804

**Published:** 2019-01-25

**Authors:** Qian Jiang, Li Cheng, Daiyuan Ma, Yanli Zhao

**Affiliations:** 1 Department of Oncology, Affiliated Hospital of North Sichuan Medical College, Nanchong, Sichuan 637000, China; 2 Basic Medical College, Chengdu medical college, Chengdu, Sichuan 610500, China

**Keywords:** angiogenesis, FBXL19-AS1, lung cancer, miR-431-5p, progression, RAF1

## Abstract

Lung cancer is the leading cause of cancer-related mortality worldwide, characterized by uncontrolled proliferation and metastasis of lung cancer cells. Tumor angiogenesis plays a key role in proliferation and metastasis in cancers, and is an essential component in microenvironment. It has been reported that long non-coding RNA FBXL19-AS1 plays an oncogenic role in colorectal cancer. However, the molecular mechanism of FBXL19-AS1 in lung cancer has not been fully elucidated. In the present study, we found that FBXL19-AS1 expression was up-regulated in lung cancer tissues and cell lines. FBXL19-AS1 knockdown inhibited cell proliferation, migration, invasion, and angiogenesis in lung cancer cells. Molecular mechanism exploration uncovered that FBXL19-AS1 acted as a molecular sponge of miR-431-5p and that RAF1 was a downstream target of miR-431-5p in lung cancer. Moreover, there was a negative association between miR-431-5p expression and FBXL19-AS1 or RAF1 expression in tumor tissues. Through rescue experiments, we discovered that overexpression of RAF1 partially rescued FBXL19-AS1 knockdown-mediated inhibition of angiogenesis and progression in lung cancer. Together, these results indicated that FBXL19-AS1 was involved in progression and angiogenesis in lung cancer by targeting miR-431-5p/RAF1 axis, which provided a new insight into the therapeutic strategies of lung cancer.

## Introduction

Lung cancer is the leading cause of cancer-related mortality worldwide, with the highest incidence in males and the third in females [1]. Lung cancer accounts for more than 1/4 of all cancer deaths, with only 10–15% of 5-year overall survival [2]. Tumor angiogenesis plays a key role in cancers, and it is an essential component in microenvironment of tumor growth and metastasis [3]. Tumor blood vessels provide oxygen and nutrients for the metabolism of tumor tissues, allowing rapid growth of tumor and also providing a base for distant metastasis of the tumor [4]. There are many studies on various biomarkers in lung cancer, but the potential molecular regulation mechanisms of these biomarkers are still not very clear. Therefore, it is greatly important to find out the biological targets related to anti-angiogenesis in lung cancer and better understand the molecular mechanism of their regulation.

Long non-coding RNA (lncRNA) is a class of non-coding RNA with a length of more than 200 nucleotides. Accumulating evidence confirm that lncRNAs emerge as a major regulator of inflammation diseases and cancers [5–8]. Increasing researchers has dedicated themselves to revealing the function of lncRNAs in cancers. For example, long non-coding RNA CPS1-IT1 suppresses cell proliferation and metastasis in human lung cancer [9]. AFAP1-AS1 is up-regulated in lung cancer and promotes invasion and metastasis [10]. Some lncRNAs can also participate in the regulation of angiogenesis in cancers. Long non-coding RNA JHDM1D-AS1 promotes tumor growth by regulating angiogenesis in response to nutrient starvation [11]. LincRNA-p21 affects prognosis in resected non-small-cell lung cancer patients through angiogenesis regulation [12]. A recent study found that long non-coding RNA FBXL19-AS1 plays oncogenic role in colorectal cancer by sponging miR-203 [13]. Nevertheless, the biological functions and molecular mechanisms of lncRNA FBXL19-AS1 in angiogenesis of lung cancer are unclear.

In the present study, we planned to investigate whether lncRNA FBXL19-AS1 exerted the oncogenic role in angiogenesis and progression of lung cancer via miR-431-5p/RAF1 axis. First, we determined the expression level of FBXL19-AS1 in lung cancer tissues and cells, and investigated the biological function of FBXL19-AS1 through function assays. Subsequently, we predicted and confirmed the interaction between miR-431-5p and lncRNA FBXL19-AS1. Additionally, we further identified RAF1 as a downstream target for miR-431-5p. Finally, we proved that FBXL19-AS1 regulated angiogenesis and tumor progression in lung cancer through miR-431-5p/RAF1 axis.

## Materials and methods

### Clinical samples

A total of 84 paired tumor tissues and adjacent non-tumor tissues were obtained from patients with lung cancer at Affiliated Hospital of North Sichuan Medical College. All specimens were immediately stored at −80°C. These patients received no other treatment prior to surgery. All written informed consents were signed by patients before surgery, and this work was approved by the Ethics Committee of Affiliated Hospital of North Sichuan Medical College.

### Cell culture and transfection

Lung cancer cell lines (A549, H1975, SPC-A-1, H125, and H1299) and normal human lung cells (MRC-5) were provided by the American Type Culture Collection (ATCC; Manassas, VA, U.S.A.). All cells were cultured in Roswell Park Memorial Institute (RPMI) 1640 medium supplemented with 10% fetal bovine serum (FBS; Gibco/Invitrogen Inc., Carlsbad, CA, U.S.A.), streptomycin (100 mg/ml), and penicillin (100 U/ml), and then they were maintained at 37°C in humidified atmosphere with 5% CO_2_.

For down-regulation of FBXL19-AS1 in cells, short hairpin RNA (shRNA) specifically targeting FBXL19-AS1 were designed and synthesized by GenePharma (Shanghai, China). The full-length sequences of FBXL19-AS1 and RAF1 were respectively synthesized and subcloned into pcDNA3.1 (Invitrogen, Carlsbad, U.S.A.) plasmid to produce pcDNA3.1/FBXL19-AS1 and pcDNA3.1/RAF1. MiR-431-5p mimic, miR-431-5p inhibitor, and the corresponding negative controls (miR-NC) were purchased from GenePharma. All plasmids were transfected into A549 and H1299 cells by using Lipofectamine 2000 (Invitrogen, Carlsbad, CA, U.S.A.) in reference of manufacturer’s recommendations. The sequences of shRNA were:

sh-FBXL19-AS1-1: 5′-CCG GCC TCC CTA AGT GTT GGG ATT ACT CGA GTA ATC CCA ACA CTT AGG GAG GTT TTT TG-3′;

sh-FBXL19-AS1-2: 5′-CCG GGC ATT TAA TTT GGC ATA GCA ACT CGA GTT GCT ATG CCA AAT TAA ATG CTT TTT TG-3′.

### RNA extraction and quantitative real-time PCR

Total RNA were extracted from A549 or H1299 cells with the employment of Trizol reagent (Takara, Otsu, Japan). RNAs were reverse transcribed into cDNA by using TaqMan™ Advanced miRNA cDNA Synthesis Kit (Waltham, MA, U.S.A.) or the reverse transcription kit (Takara, Otsu, Japan). The RT-qPCR was conducted by SYBR Green PCR Kit (Takara, Otsu, Japan). Internal controls were GAPDH and U6. The results of RT-qPCR were analyzed by using Applied Biosystems Step One Plus Real-Time PCR System (Applied Biosystems, Foster city, U.S.A.), and the 2^−^^ΔΔ*C*^_t_ method was used to examine these relative expression levels. The primers for RT-qPCR were as follows: FBXL19-AS1: 5′-GGT ACA ACT ACG GAT ATG A-3′ (Forward) and 5′-TAC GTC TCG ACC ATT ACG CA-3′ (Reverse); miR-431-5p: 5′-TGT CTT GCA GGC CGT CAT G-3′ (Forward) and 5′-GCT GTC AAC GAT ACG CTA CCT A-3′ (Reverse); RAF1: 5′-GGG AGC TTG GAA GAC GAT CAG-3′ (Forward) and 5′-ACA CGG ATA GTG TTG CTT GTC-3′ (Reverse); GAPDH: 5′-GAA GGT GAA GGT CGG AGT C-3′ (Forward) and 5′-GAA GAT GGT GAT GGG ATT TC-3′ (Reverse); U6: 5′-ATT GGA ACG ATA CAG AGA AGA TT-3′ (Forward) and 5′-GGA ACG CTT CAC GAA TTT G-3′ (Reverse).

### CCK-8 assay

Cell-Counting Kit 8 (CCK8; Dojindo Molecular Technologies) was used to measure the proliferation of lung cancer cells. The transfected cells (1 × 10^4^ cells/well) were seeded in 96-well plates. After 24, 48, 72 and 96 h of incubation, CCK-8 solution (10 ml) was added to each well, and then the incubation continued for 4 h. The absorbance at 450 nm was detected by using a Multiskan Go spectrophotometer (Thermo Fisher Scientific, Inc.).

### Colony formation assay

Transfected cells (1 × 10^3^ cells/well) were plated into six-well plates and maintained in RPMI 1640 medium. Replace the medium every 3 days. After 14 days, colonies were fixed by the use of methanol and stained by the use of 0.1% crystal violet. Then, colonies were counted manually.

### Transwell assay

Transwell assay was used to examine cell invasion and migration. Transwell chambers (Corning Incorporated, Corning, NY, U.S.A.) with (for invasion assay) or without (for migration assay) matrigel (BD Biosciences, Bedford, MA, U.S.A.) were used for transwell assay. Two hundred microliters of RPMI 1640 medium containing transfected cells (1 × 10^4^ cells/well) was added into the upper chambers, and 800 μl of RPMI 1640 medium containing 10% FBS was added into the lower chambers. After 48 h of incubation, invaded or migrated cells were fixed with the application of methanol and stained utilizing 0.5% crystal violet (Amresco Co., Solon, OH, U.S.A.). Then, stained cells were counted under a light microscope (Olympus Corporation, Tokyo, Japan).

### RIP assay

RIP assay was performed by using Magna RNA-binding protein immunoprecipitation kit (Millipore, Billerica, MA, U.S.A.). Cell lysate (A549 and H1299) was incubated in RIP buffer containing magnetic beads that were conjugated with human anti-Ago2 antibody. Input and normal IgG were used as controls. Proteinase K was used to isolate immunoprecipitated RNAs. Then, purified RNAs were detected by RT-qPCR.

### Pull-down assay

Pull-down assay was used to detect the potential binding capacity of FBXL19-AS1 and miR-431-5p. FBXL19-AS1-Mt, FBXL19-AS1-Mut, and NC were biotinylated to be Bio-FBXL19-AS1-Mt, Bio-FBXL19-AS1-Mut, and Bio-NC by GenePharma Company (Shanghai, China). Bio-FBXL19-AS1-Mt, Bio-FBXL19-AS1-Mut, and Bio-NC were transfected into A549 or H1299 cells. After incubation of 48 h, cells were lysed, and the cell lysate was incubated with Dynabeads M-280 Streptavidin (Invitrogen, CA). Purified RNA complex was measured by RT-qPCR.

Pull-down assay was used to detect the potential binding capacity of RAF1 and miR-431-5p. MiR-431-5p-Wt, miR-431-5p-Mut, and miR-NC were transcribed employing Transcript Aid T7 High Yield Transcription Kit (ThermoFisher Scientific, U.S.A.). Bio-miR-431-5p-Wt, Bio-miR-431-5p-Mut, and Bio-miR-NC were produced employing Biotin RNA labeling mix (Roche Diagnostics, Indianapolis, IN, U.S.A.). Two hundred micrograms of cell lysates (A549 and H1299) and 50 pmol biotinylated RNA were mixed, and then incubated with 50 μl streptavidin agarose beads (Invitrogen, Carlsbad, CA, U.S.A.) at 4°C for 1 h. After washing, the proteins that were bounded with RNA were eluted by using elution buffer, and then the protein level of RAF1 was measured by Western blotting.

### Luciferase reporter assay

The 3′-UTR sequences of RAF1 containing the predicted miR-431-5p binding sites and full-length sequences of FBXL19-AS1 were subcloned into the pGL3 vector (Promega, Madison, WI, U.S.A.) to produce the wild-type RAF1 reporter (RAF1-Wt) and the wild-type FBXL19-AS1 reporter (FBXL19-AS1-Wt). The mutant-type FBXL19-AS1 reporter (FBXL19-AS1-Mut) and the mutant-type RAF1 reporter (RAF1-Mut) were provided by GeneArt™ Site-Directed Mutagenesis System (Thermo Fisher Scientific). MiR-431-5p mimic, miR-431-5p inhibitor, or miR-NC were co-transfected with FBXL19-AS1-Wt or FBXL19-AS1-Mut into A549 or H1299 cells by the use of Lipofectamine 2000. MiR-NC, miR-431-5p mimic, or miR-431-5p mimic+pcDNA3.1/FBXL19-AS1 were also co-transfected with RAF1-Wt or RAF1-Mut into A549 or H1299 cells. After 48 h of incubation, the luciferase activities were measured by the use of luciferase reporter assay system (Promega, Madison WI, U.S.A.).

### Western blot

Proteins were extracted by the use of RIPA lysis buffer (Beyotime Biotechnology, China) supplemented with protease inhibitors (Roche, China). Then these proteins were quantified with the employment of BCA™ Protein Assay Kit (Pierce, Appleton, U.S.A.). Proteins were separated through sodium dodecyl sulfate-polyacrylamide gel electrophoresis, and then electrophoretically transferred onto the polyvinylidene difluoride membranes. After blocking with skim milk, membranes were incubated with the primary antibodies overnight at 4°C. The primary antibodies to RAF1, VEGF, Ang1, FGF2, and GAPDH were all purchased from Abcam Company (Abcam, Cambridge, U.K.). After that, these membranes were then incubated with secondary antibody for 2 h at room temperature. The signals were captured via the use of chemiluminescent detection system. Internal control was GADPH.

### Statistical analysis

Data were shown as the mean ± standard deviation (SD). Each experiment was repeated three times. The one-way ANOVA or Student’s *t*-test were used to compare differences among groups. SPSS 20.0 software (SPSS, Chicago, IL, U.S.A.) was used for statistical analysis. Kaplan Meier and Log-rank test were used to carry out survival analysis. Spearman’s correlation analysis was used to determine correlation examination. Any value of *P*<0.05 was considered statistically significant.

## Results

### FBXL19-AS1 expression is up-regulated in lung cancer tissues and cell lines and high level of FBXL19-AS1 predicts poor prognosis

**Figure 1:**
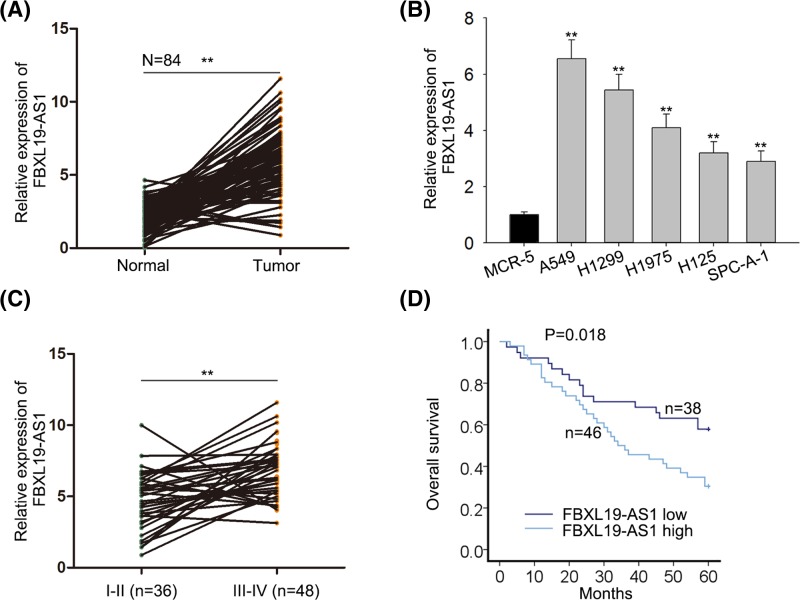
FBXL19-AS1 is up-regulated in lung cancer tissues and cell lines and relates to poor prognosis of patients (**A**) RT-qPCR results showed that expression of FBXL19-AS1 was higher in tumor tissues than in adjacent non-tumor tissues. (**B**) RT-qPCR results showed that expression of FBXL19-AS1 was higher in lung cancer cell lines (A549, H1299, H1975, H125, and SPC-A-1) than in normal human lung cells (MRC-5). (**C**) Relative FBXL19-AS1 expression levels in different clinical stages (I-II and III-IV). (**D**) Kaplan–Meier curve and the log-rank test showed that the expression of FBXL19-AS1 was associated with overall survival in patients with lung cancer. Error bars represent the mean ± SD of at least three independent experiments. **P<0.01 versus control group.

To explore the roles of lncRNA FBXL19-AS1 in lung cancer, we first detected the relative expression of FBXL19-AS1 in 84 paired lung cancer tissues and cells. The results showed that FBXL19-AS1 expression was markedly up-regulated in lung cancer tissues compared with adjacent normal tissues (Figure 1A). There was a remarkable increase in FBXL19-AS1 level in lung cancer cell lines (A549, H1299, H1975, H125, SPC-A-1) when compared with that in the normal human lung cells (MCR-5) (Figure 1B). Moreover, we explored the correlation between the expression of FBXL19-AS1 and the clinical characteristic of lung cancer patients. It was revealed that the high expression of FBXL19-AS1 was associated with tumor size, differentiation, TNM stage, and lymph node metastasis (Table 1, *P*<0.05). Increased expression of FBXL19-AS1 was also observed in advanced tumor stage patients (Figure 1C). Furthermore, the patients who had high levels of FBXL19-AS1 came out with notably poorer prognosis than those who had low levels of FBXL19-AS1 (Figure 1D). Multivariate analysis showed that only lncRNA FBXL19-AS1 expression (*P*=0.003) and TNM stage (*P*=0.014) were independent prognostic factors for lung cancer patients (Table 2). In conclusion, the results above indicated that FBXL19-AS1 may be an oncogene related to tumor formation and poor prognosis in lung cancer.

**Table 1:** Correlation between FBXL19-AS1 expression and clinical features (*n*=84)

Variable	FBXL19-AS1 expression		*P*-value
	Low	High	
**Age**			
<60	21	27	0.826
≥60	17	19	
**Gender**			
Male	22	28	0.826
Female	16	18	
**Tumor Size**			
<3cm	25	17	0.015*
≥3cm	13	29	
**Histology**			
Adenoma	21	28	0.660
Squamous	17	18	
**Differentiation**			
Well	23	13	0.004*
Moderate-poor	15	33	
**TNM stage**			
I-II	22	14	0.015*
III-IV	16	32	
**Lymph node metastasis**			
No	26	14	0.001*
Yes	12	32	

Low/high by the sample median. Pearson χ^2^ test. *P<0.05 was considered statistically significant.

**Table 2:** Multivariate analysis of prognostic parameters in patients with lung cancer by Cox regression analysis

Variable	Category	*P*-value
Age		
	<60	0.595
	≤60	
Gender		
	Male	0.519
	Female	
Tumor size		
	<3 cm	0.982
	≥3 cm	
Histology		
	Adenoma	0.978
	Squamous	
Differentiation		
	Well	0.726
	Moderate-poor	
TNM Stage		
	I-II	0.014*
	III-IV	
Lymph node metastasis		
	No	0.122
FBXL19-AS1 level	Yes	0.003*
	Low	
	High	

Proportional hazards method analysis showed a positive, independent prognostic importance of FBXL19-AS1 expression (*P*=0.003). *P<0.05 was considered statistically significant.

### FBXL19-AS1 knockdown inhibits proliferation, migration, invasion, and angiogenesis in lung cancer cells

**Figure 2:**
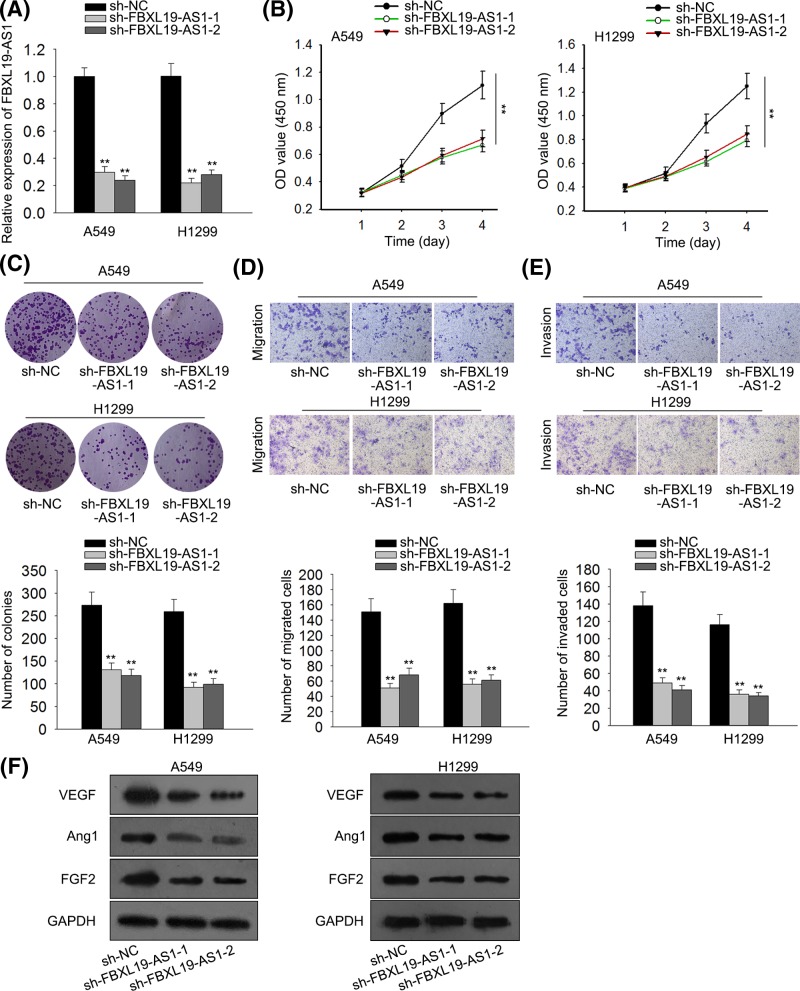
FBXL19-AS1 knockdown inhibits proliferation, migration and invasion, and reduces the expression levels of angiogenesis related proteins in lung cancer cells (**A**) The knockdown efficiency of FBXL19-AS1 in A549 and H1299 cells was examined by RT-qPCR. (**B**) At the indicated time points (24, 48, 72 and 96 h), the proliferation of transfected cells was determined by CCK8 assay. (**C**) After 2 weeks of transfection, colony formation assay was performed. (**D** and **E**) Transwell assay presented that the knockdown of FBXL19-AS1 inhibits migration and invasion in A549 and H1299 cells. (**F**) After 48 h of transfection, protein expressions of VEGF, Ang1, and FGF2 were measured by Western blot assay. Error bars represent the mean ± SD of at least three independent experiments. **P<0.01 versus control group.

In order to investigate the possible biological role of FBXL19-AS1 in lung cancer tumorigenesis, we knocked down FBXL19-AS1 using sh-FBXL19-AS1-1 or sh-FBXL19-AS1-2 with sh-NC as scramble control. Then, the knockdown efficiency of sh-FBXL19-AS1 in A549 and H1299 cells was detected by RT-qPCR assay. As shown in Figure 2A, the introduction of sh-FBXL19-AS1-1/2 caused a significant reduction in FBXL19-AS1 levels in A549 and H1299 cells compared with scramble control, suggesting that sh-FBXL19-AS1-1/2 could be used for the subsequent loss-of-function experiments. Next, the effects of FBXL19-AS1 down-regulation on cell proliferation, migration, invasion, and angiogenesis were further examined in A549 and H1299 cells. CCK8 assay revealed that proliferation ability was significantly declined in FBXL19-AS1-depleted A549 and H1299 cells than that in mock cells (Figure 2B). Colony formation assay showed that FBXL19-AS1 knockdown triggered a significant reduction of colony numbers in A549 and H1299 cells (Figure 2C). Transwell assay further manifested that knockdown of FBXL19-AS1 inhibited the migration and invasion of A549 and H1299 cells (Figure 2D,E). Western blot assay showed that FBXL19-AS1 deficiency reduced the expression of angiogenesis associated proteins (VEGF, Ang1, and FGF2) in A549 and H1299 cells (Figure 2F). All these data suggested that down-regulation of FBXL19-AS1 inhibits proliferation, migration, invasion, and angiogenesis in lung cancer cells.

### FBXL19-AS1 acts as a molecular sponge of miR-431-5p in lung cancer cells

**Figure 3:**
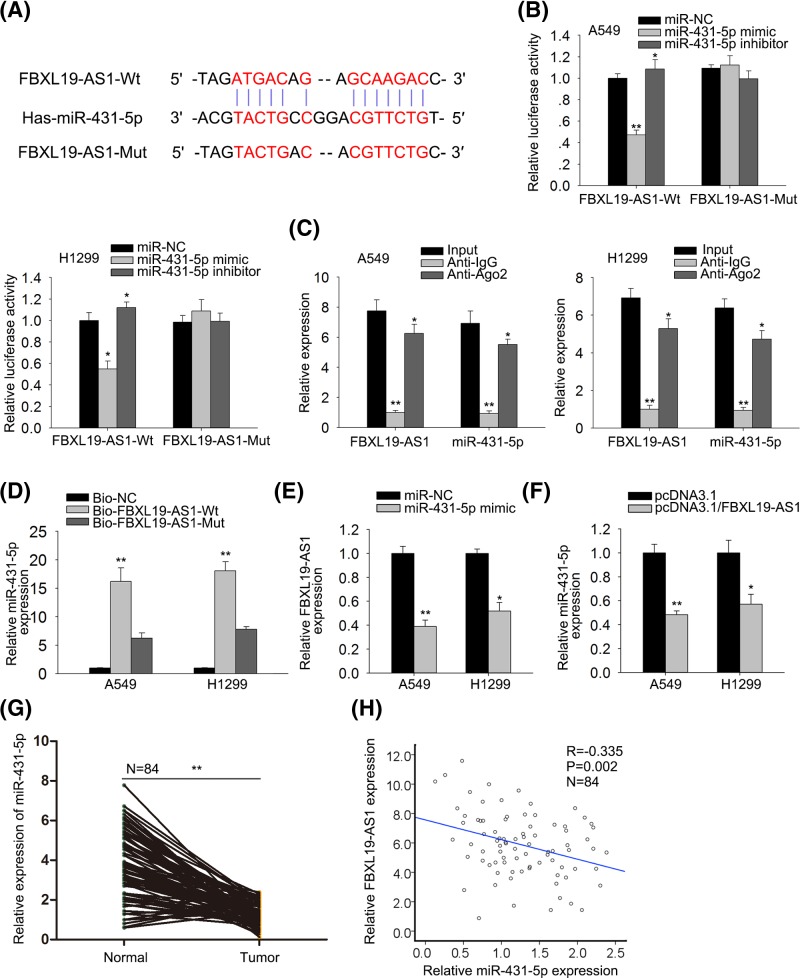
FBXL19-AS1 acts as a sponge of miR-431-5p in lung cancer (**A**) The predicted binding sites of FBXL19-AS1 and miR-431-5p, and the mutant sites in mutant-type FBXL19-AS1 reporter. (**B**) FBXL19-AS1 (Wt) or FBXL19-AS1 (Mut) reporter were co-transfected with miR-NC, miR-431-5p mimic, or miR-431-5p inhibitor into A549 and H1299 cells. After 48 h, relative luciferase activity was detected by using luciferase reporter assay. (**C**) RIP and RT-qPCR assays were performed to determine the enrichment degrees of FBXL19-AS1 and miR-431-5p in IgG or Ago2 immunoprecipitate. The whole sample was divided in four, with 10% as input, 30% as positive control, 30% as negative control, and 30% as IP sample. (**D**) RNA pull-down assay was performed to further confirm the binding ability between FBXL19-AS1 and miR-431-5p in A549 and H1299 cells. (**E**) RT-qPCR results showed that overexpression of miR-431-5p reduced the expression of FBXL19-AS1. (**F**) RT-qPCR results showed that overexpression of FBXL19-AS1 reduced the expression of miR-431-5p. (**G**) RT-qPCR results showed that expression of miR-431-5p was lower in tumor tissues than in adjacent non-tumor tissues. (**H**) Spearman’s correlation analysis showed the negative correlation between expression of FBXL19-AS1 and miR-431-5p. Error bars represent the mean ± SD of at least three independent experiments. *P<0.05, **P< 0.01 versus control group.

We then investigated the regulatory mechanism of lncRNA FBXL19-AS1 in lung cancer. Abundant evidence propose that lncRNAs could act as ceRNAs to regulate target gene expression by sponging miRNAs [14]. Therefore, we suspected that lncRNA FBXL19-AS1 in lung cancer also played a role in this way. We searched starBase and targetSites online websites and chose miR-431-5p from the putative targets of FBXL19-AS1 (Figure 3A) because miR-431-5p has been reported to have antitumor effect in multiple cancers [15,16]. Then we validated the interaction of FBXL19-AS1 and miR-431-5p. Luciferase assay showed that luciferase activity of wild-type FBXL19-AS1 was significantly decreased in miR-431-5p mimic transfected cells, but was markedly increased in cells after introduction of miR-431-5p inhibitor (Figure 3B). However, no significant change of luciferase activity was found in groups of mutant-type FBXL19-AS1, indicating that FBXL19-AS1 interacted with miR-431-5p. RIP assay results showed that FBXL19-AS1 and miR-431-5p were co-immunoprecipitated by the anti-Ago2 antibody but not the IgG antibody (Figure 3C). Pulldown assay further confirmed that miR-431-5p could directly bind with FBXL19-AS1 (Figure 3D). Additionally, we studied the correlation between FBXL19-AS1 and miR-431-5p, finding through RT-qPCR that miR-431-5p overexpression reduced FBXL19-AS1 level, and vice versa (Figure 3E,F). The expression of miR-431-5p was significantly lower in tumor tissues than that in adjacent non-tumor tissues (Figure 3G). Moreover, Spearman’s correlation analysis demonstrated the negative correlation between FBXL19-AS1 and miR-431-5p expression in tumor tissues (*r* = −0.335, *P*<0.05, Figure 3H). Taken together, these findings suggested that FBXL19-AS1 acts as a molecular sponge of miR-431-5p in lung cancer cells.

### RAF1 is a downstream target of miR-431-5p

**Figure 4:**
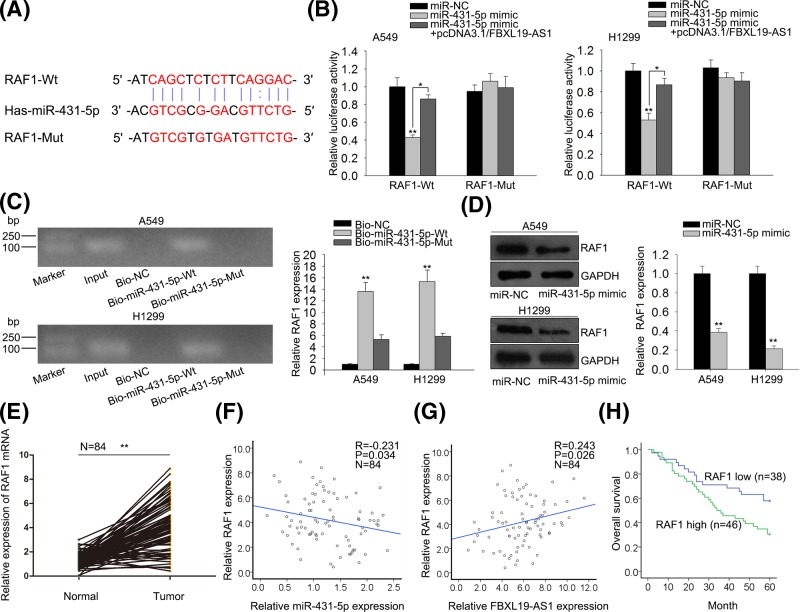
MiR-431-5p directly targets RAF1 and is negatively correlated to RAF1 expression (**A**) The predicted binding sites of miR-431-5p and RAF1 3′-UTR, and mutant sites in mutant-type RAF1 reporter. (**B**) RAF1 (Wt) or RAF1 (Mut) reporter were co-transfected with miR-NC, miR-431-5p mimic or miR-431-5p mimic +pcDNA3.1/FBXL19-AS1 into A549 and H1299 cells. After 48 h, relative luciferase activity was detected by using luciferase reporter assay. (**C**) RNA pull-down assay was performed to further confirm the binding ability between RAF1 and miR-431-5p in A549 and H1299 cells. (**D**) After 48 h of transfection, the protein expression of RAF1 was measured by western blot assay. (**E**) RT-qPCR results showed that overexpression of RAF1 reduced the expression of miR-431-5p. (**F**) RT-qPCR results showed that expression of RAF1 was higher in tumor tissues than in adjacent non-tumor tissues. (**G**) Spearman’s correlation analysis demonstrated the negative correlation between expression of RAF1 and miR-431-5p. (**H**) Spearman’s correlation analysis showed the positive correlation between expression of FBXL19-AS1 and RAF1. Error bars represent the mean ± SD of at least three independent experiments. *P<0.05, **P<0.01 versus control group.

To find out the downstream target of miR-431-5p, we then predicted the potential target genes of miR-431-5p by using RNA22, GRCh37, and miRBase. Bioinformatics instruments showed the 3′-UTR of RAF1 included the targeting site of miR-431-5p (Figure 4A). RAF1 has been reported to have oncogenic effect in human cancers and its relationship with tumor angiogenesis has also been revealed [17–19]. Luciferase assay revealed that luciferase activity of wild-type RAF1 was significantly decreased in miR-431-5p mimic transfected cells, but increased after co-transfection of pcDNA3.1/FBXL19-AS1, indicating that RAF1 could bind with miR-431-5p and that FBXL19-AS1 could regulate RAF1 expression by targeting miR-431-5p (Figure 4B). However, no significant difference was found in luciferase activity among mutant-type RAF1 groups. Pull-down assay further confirmed that miR-431-5p could directly bind with RAF1 (Figure 4C). Fatherly, we discovered that RAF1 expression was down-regulated in miR-431-5p mimic transfected lung cancer cells by Western blot (Figure 4D). Moreover, the expression level of RAF1 was significantly up-regulated in lung cancer tissues compared with non-tumor tissues (Figure 4E). Moreover, Spearman’s correlation analysis demonstrated that there was the negative correlation between expression of RAF1 and miR-431-5p in tumor tissues, and the positive correlation between expression of FBXL19-AS1 and RAF1 in tumor tissues (*r* = −0.231, *r* = 0.243, *P*<0.05, Figure 4F,G). The patients who had high levels of RAF1 came out with notably poorer prognosis than those who had low levels of RAF1 (Figure 4H). All these data suggested that RAF1 is a downstream target of miR-431-5p, and FBXL19-AS1 could regulate RAF1 expression by targeting miR-431-5p.

### FBXL19-AS1 promotes lung cancer angiogenesis and progression via regulating RAF1

**Figure 5:**
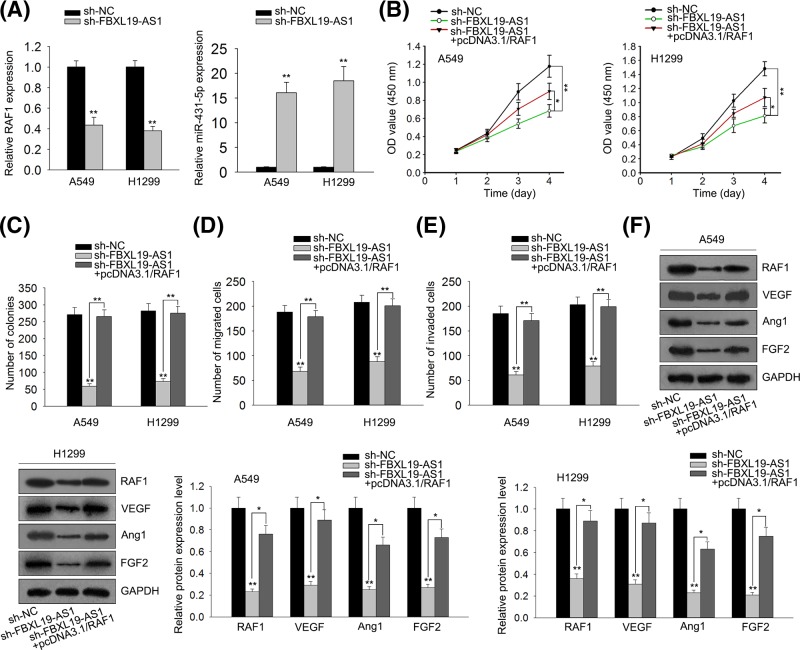
Overexpression of RAF1 weakens FBXL19-AS1 knockdown-mediated inhibition of lung cancer progression (**A**) RT-qPCR results showed that down-expression of FBXL19-AS1 reduced the expression of RAF1. (**B**) CCK8 assay implied that decreased FBXL19-AS1 expression inhibited cell proliferation in A549 and H1299 cells. However, overexpression of RAF1 weakened FBXL19-AS1 knockdown-mediated inhibition of cell proliferation. (**C**) Colony formation assay further implied that decreased FBXL19-AS1 expression inhibited cell proliferation in A549 and H1299 cells. However, overexpression of RAF1 weakened FBXL19-AS1 knockdown-mediated inhibition of cell proliferation. (**D** and **E**) Transwell assay presented that decreased FBXL19-AS1 expression inhibited cells migration and invasion in A549 and H1299 cells. However, overexpression of RAF1 weakened FBXL19-AS1 knockdown-mediated inhibition of cells migration and invasion. (**F**) Western blot assay presented that decreased FBXL19-AS1 expression inhibited protein expressions of VEGF, Ang1, and FGF2 in A549 and H1299 cells. However, overexpression of RAF1 weakened FBXL19-AS1 knockdown-mediated inhibition of protein expressions (VEGF, Ang1, and FGF2). Error bars represent the mean ± SD of at least three independent experiments. *P<0.05, **P<0.01 versus control group.

Finally, we investigated whether FBXL19-AS1 promotes the angiogenesis and progression by regulating RAF1. We discovered that RAF1 expression was down-regulated in sh-FBXL19-AS1 transfected cells (Figure 5A). Then, the co-transfection of pcDNA3.1/RAF1 reversed the inhibitory roles of sh-FBXL19-AS1 in the proliferation, migration, and invasion of lung cancer cells (Figure 5B–E). Furthermore, compared with sh-FBXL19-AS1 transfected cells, the expression of angiogenesis associated proteins (VEGF, Ang1, and FGF2) also presented a regain in sh-FBXL19-AS1 and pcDNA3.1/RAF1 co-transfected cells (Figure 5F). RAF1 overexpression partially rescued FBXL19-AS1 knockdown-mediated inhibition of lung cancer angiogenesis and progression. All the above results led to the conclusion that knockdown of long non-coding RNA FBXL19-AS1 inhibits proliferation, migration, invasion, and angiogenesis in lung cancer by targeting miR-431-5p/RAF1.

## Discussion

Previous studies revealed that lncRNAs participate in many cancers, such as gastric cancer, bladder cancer, and lung cancer [20–22]. It has been reported that long non-coding RNA FBXL19-AS1 plays oncogenic role in colorectal cancer [13]. Nevertheless, the biological functions and molecular mechanisms of lncRNA FBXL19-AS1 in lung cancer are unclear. In the present study, we found that the relative expression of FBXL19-AS1 in tumor tissues were significantly higher than that in adjacent non-tumor tissues and cell lines. Knockdown of FBXL19-AS1 reduced the proliferation, migration, and invasion in A549 and H1299 cell lines. What’s more, we discovered that knockdown of FBXL19-AS1 reduced the expression of crucial angiogenesis factors (VEGF, Ang1, and FGF2). All these data indicated that FBXL19-AS1 promoted the angiogenesis and progression of lung cancer.

MicroRNAs (miRNAs) are short non-coding RNA molecules with 20–24 nucleotides, and play important roles in the post-transcriptional regulation of gene expression [23,24]. Recently, it has been reported that lncRNAs act as ‘sponges’ to bind with specific miRNAs and then regulate multiple diseases [25,26]. For example, the lncRNA HOTAIR functions as a ceRNA to increase the expression of HER2 via miR-331-3p and to drive gastric cancer growth and invasion [27]. LncRNA NNT-AS1 promotes the proliferation, and invasion of lung cancer cells via regulating miR-129-5p expression [28]. It has been reported that long non-coding RNA FBXL19-AS1 plays oncogenic role in colorectal cancer by sponging miR-203 [13]. But the interaction of FBXL19-AS1 with target miRNAs in lung cancer cells was still not clear. In our present study, we selected miR-431-5p from the potential target miRNAs for FBXL19-AS1 on miRcode and starBase online websites. MiR-431-5p has been reported to have antitumor effect in multiple cancers [15,16]. In the present study, we further confirmed the interaction between FBXL19-AS1 and miR-431-5p as well as their negative correlation in lung cancer. All these data indicated that FBXL19-AS1 regulated lung cancer progression by sponging miR-431-5p.

RAF1 has been reported to play a carcinogenic role in human cancers and is related to tumor angiogenesis [17–19]. Inhibition of RAF1 kinase activity restores apicobasal polarity and impairs tumor growth in human colorectal cancer [17]. MiR-7-5p inhibits vascular endothelial cell proliferation by targeting RAF1 [19]. Present study predicted according to bioinformatics analysis that miR-431-5p could bind to RAF1. What’s more, we found that down-regulation of miR-431-5p as well as up-regulation of FBXL19-AS1 can increase RAF1 expression. Finally, rescue assays delineated that overexpression of RAF1 partially rescued FBXL19-AS1 knockdown-mediated inhibition of lung cancer progression and the expression of angiogenesis associated proteins.

In summary, our study proved that FBXL19-AS1 silencing suppressed the progression and angiogenesis of lung cancer progression by targeting miR-431-5p/RAF1, indicating the potential of FBXL19-AS1/miR-431-5p/RAF1 axis as a new biological diagnostic/therapeutic target for angiogenesis of lung cancer to improve prognosis.

## Supporting information

**Supplemental Table S1:** 

## Abbreviations

lncRNA: long non-coding RNA
RPMI 1640: Roswell Park Memorial Institute
shRNA: short hairpin RNA
