# Supplementary material for: FBXL19-AS1 exerts oncogenic function by sponging miR-431-5p to regulate RAF1 expression in lung cancer
Source: Biosci Rep. 2019 Jan 25;39(1):BSR20181804. doi: 10.1042/BSR20181804 (PMC6350044; doi:10.1042/BSR20181804)
Supplement: Supplementary file 1 [file BSR-2018-1804_supp.pdf]

| Target gene | Representative transcript | Gene name | Representative miRNA |
| --- | --- | --- | --- |
| CD44 | ENST00000278386.6 | CD44 molecule (Indian blood group) | hsa-miR-431-5p |
| DSCR4 | ENST00000398948.1 | Down syndrome critical region gene 4 | hsa-miR-431-5p |
| EGFR | ENST00000275493.2 | epidermal growth factor receptor | hsa-miR-431-5p |
| CD274 | ENST00000381573.4 | CD274 molecule | hsa-miR-431-5p |
| RPS15A | ENST00000576436.1 | ribosomal protein S15a | hsa-miR-431-5p |
| CD34 | ENST00000356522.4 | CD34 molecule | hsa-miR-431-5p |
| ZEB1 | ENST00000361642.5 | zinc finger E-box binding homeobox 1 | hsa-miR-431-5p |
| SMAD4 | ENST00000398417.2 | SMAD family member 4 | hsa-miR-431-5p |
| HHIP | ENST00000296575.3 | hedgehog interacting protein | hsa-miR-431-5p |
| SRPX2 | ENST00000373004.3 | sushi-repeat containing protein, X-linked 2 | hsa-miR-431-5p |

| | | | |
|---|---|---|---|
| NCL | ENST00000322723.4 | nucleolin | hsa-miR-431-5p |
| NOX5 | ENST00000260364.5 | NADPH oxidase, EF-hand calcium binding domain 5 | hsa-miR-431-5p |
| EIF4E | ENST00000450253.2 | eukaryotic translation initiation factor 4E | hsa-miR-431-5p |
| TGFA | ENST00000295400.6 | transforming growth factor, alpha | hsa-miR-431-5p |
| CDK6 | ENST00000265734.4 | cyclin-dependent kinase 6 | hsa-miR-431-5p |
| CD47 | ENST00000361309.5 | CD47 molecule | hsa-miR-431-5p |
| IL8 | ENST00000307407.3 | interleukin 8 | hsa-miR-431-5p |
| RAF1 | ENST00000251849.4 | v-raf-1 murine leukemia viral oncogene homolog 1 | hsa-miR-431-5p |
| FAM65B | ENST00000259698.4 | family with sequence similarity 65, member B | hsa-miR-431-5p |

Supplementary table S1

The potential target genes associated with angiogenesis for miR-431-5p
